# Education can improve the negative perception of a threatened long-lived scavenging bird, the Andean condor

**DOI:** 10.1371/journal.pone.0185278

**Published:** 2017-09-26

**Authors:** Verónica B. Cailly Arnulphi, Sergio A. Lambertucci, Carlos E. Borghi

**Affiliations:** 1 Universidad Nacional de San Juan—CIGEOBIO CONICET, San Juan, Argentina; 2 Laboratorio Ecotono, Universidad Nacional del Comahue—INIBIOMA CONICET, Bariloche, Río Negro, Argentina; 3 Departamento de Biología y Museo de Ciencias Naturales, Facultad de Ciencias Exactas, Físicas y Naturales, Universidad Nacional de San Juan, San Juan, Argentina; Universidade de Aveiro, PORTUGAL

## Abstract

Human-wildlife conflicts currently represent one of the main conservation problems for wildlife species around the world. Vultures have serious conservation concerns, many of which are related to people's adverse perception about them due to the belief that they prey on livestock. Our aim was to assess local perception and the factors influencing people's perception of the largest scavenging bird in South America, the Andean condor. For this, we interviewed 112 people from Valle Fértil, San Juan province, a rural area of central west Argentina. Overall, people in the area mostly have an elementary education, and their most important activity is livestock rearing. The results showed that, in general, most people perceive the Andean condor as an injurious species and, in fact, some people recognize that they still kill condors. We identified two major factors that affect this perception, the education level of villagers and their relationship with livestock ranching. Our study suggests that conservation of condors and other similar scavengers depends on education programs designed to change the negative perception people have about them. Such programs should be particularly focused on ranchers since they are the ones who have the worst perception of these scavengers. We suggest that highlighting the central ecological role of scavengers and recovering their cultural value would be fundamental to reverse their persecution and their negative perception by people.

## Introduction

Human-wildlife conflicts currently represent one of the main conservation problems for wildlife species around the world [1]. These conflicts are increasing globally due to human population growth, land use changes, habitat loss and fragmentation, and climate change, among other reasons [2]. Human-wildlife conflicts encompass several species and situations worldwide, ranging from economic losses to crop [3–6] and livestock predation [7,8], disease transmission [9], and even wildlife attacks directed at humans [10,11]. Sometimes, conflicts are related to cultural or religious issues. Such is the case with snakes and bats [12,13] or owls, loathed owing to the belief that they bring misfortune or death [14–16]. Many of the species involved in this kind of conflict have serious conservation problems. In fact, the future of many threatened populations relies on the development of a mechanism that can ensure coexistence of endangered species and humans [11,17]. Therefore, understanding the different aspects of human-wildlife conflicts is crucial for species conservation [18].

The best known conflicts involve mammal predators or rodent and insect pests [19,20]. Interestingly, human conflicts with birds, and their role in conservation, have been less studied [21]. Some of the few studied examples include the conflict between legally protected raptors (e.g. *Cyrcus cyaneus*) and game bird managers in northeastern Scotland [22]; or the conflict between the crowned eagle (*Harpyhaliaetus coronatus*) and sheep rangers in central Argentina [23] both fuelled by the economic losses perceived by land managers. Conflicts with birds are not restricted to predators, since negative interactions with scavenging birds have also been documented in North America [24,25]. Scavengers are at the top of the food chain and provide important ecosystem services by removing animal debris [26,27], yet they face serious conservation problems [28,29] many of which are driven by conflicts with local people.

Here we studied the long-lasting conflict with the Andean condor (*Vultur gryphus*), an emblematic species from the Andes mountain ranges. The condor's overall conservation status has been rated as “Near Threatened” [30], and in Argentina, despite being home to some of the largest remaining populations [31], the species has been classified as “Vulnerable” [32]. Most conservation problems condors face locally are related to negative interactions with humans. Indirect interactions comprise the effects of regional infrastructure development (e.g. roads, [33,34]) and lead contamination [28], whereas direct interactions include poisoning, trapping and hunting [28,35, 36] (CE Borghi, personal observation). Human direct persecution of condors may be the single most important issue for conservation of the local populations and is supported by the widespread belief that condors prey on livestock [37–39]. Condors are regarded as a pest in several rural areas of Argentina, leading to severe conflicts with humans in areas devoted to extensive livestock ranching [37,40,41]. However, a study specifically aimed at understanding human perception of Andean condors is still lacking.

We understand “perception” as the negative or positive appreciation humans have of condors. This appreciation is determined by people’s valuation of these birds. Some people see the cultural, ecological or tourist value of condors and build a positive perception, on the other hand, some other people view condors as livestock predators and, consequently, as a cause of economic loss, so their perception is negative [42,43]. Because perception is built on the valuation each person makes of a species, it can be highly heterogeneous and influenced by socio-demographic factors such as sex, age, occupation and education level [44].

Our aim was to assess human perception of Andean condors and its underlying factors. We hypothesized that the deeply-rooted belief that scavengers prey heavily on livestock elicits a negative perception that leads to persecution. Moreover, we expect this perception to differ depending on demographic and social factors such as gender, age, occupation, and education, as well as on people’s relationship with livestock. To test this, we interviewed people from villages immersed in the home range of the Andean condor in a rural area in Argentina’s Midwest. In this area most people have only elementary education, and their most important activity is livestock rearing.

## Materials and methods

### Ethics statement

This study was approved by the Comité de Bioética from the department of Biology of the Universidad Nacional de San Juan, Acta N°17, Exp. 02-3243-C.

### Study species / condor status

The Andean condor is a bird exclusive to the west of South America and the largest scavenging bird (13 kg in mass, 3 m in wingspan and 1.3 m in height; [35]). It is found at up to 5,000 m across the Andean mountains, extending its distribution in the South Cone to the east of the mountains of Central Argentina [45]. Condors feed mainly on carcasses of medium- to large-sized vertebrates [46], playing a fundamental ecological role in energy flow and environmental cleaning [47]. They have held important cultural value to both pre-Hispanic [37,48] and recent populations [49].

The condor’s overall conservation status vary throughout South America, but its population trend is decreasing. Its total population is estimated to number at least 10,000 individuals, roughly equivalent to 6,700 mature individuals. Since 2000, declines have continued in Ecuador (c.100 birds remain in five disjunct populations), Peru (c.120 birds in north of the country) and Bolivia (c.250 in one mountain location [50,51]).The population in Venezuela consists of nearly 30 individuals or fewer, and the one in Colombia is estimated at approximately 100 individuals [30]. The population in northern Argentina remains numerous, and in Chile, the condor population is estimated at around 2,200 birds [52]. The largest known population in Argentina is in north-western Patagonia and comprises an estimated c.300 individuals, of which c.200 are adults [31], nevertheless, the species has been classified as “Vulnerable” [32] due to the large number of conservation problems it face [45,53].

### Study area

This study was carried out in the Valle Fértil department (30° 54’ S-67°17’ W), San Juan province, Argentina. This area covers almost 700 000 ha and has approximately 7000 inhabitants [54]. Currently, extensive cattle ranching is a common and important cultural and economic practice, with herds amounting to approximately 15 000 head including cattle and goats. Condors fly, roost and feed in the western mountains of the Department (ca. 2100 m asl), and are commonly seen flying over towns. The Valle Fértil department encompasses two protected areas that occupy 44.34% of its land surface: Ischigualasto Provincial Park, a World Human Heritage Site (UNESCO), which extends over 63 000 ha and is home to a population of at least 62 condors [55] and Valle Fértil Natural Park, which covers 221 608 ha, but there is no information about the size of the condor population in this area.

### Background information on Valle Fértil villagers

The native American people that lived in Valle Fértil were Diaguita descendants called “Yacampis”. Yacampis began to disappear in the 17th century, and around 1810 only a mestizo population of Hispanic and Amerindian ancestry remained in this area. These mestizo people used the area’s wildlife, and also introduced livestock. The largest number of livestock was reached in 1908 when there were more than 74 000 head of cattle and about 255 000 goats. Horses, pigs and mules also reached their peak in this period [56]. Currently, extensive livestock ranching is still a common and important cultural and economic practice across the entire department, and the people in Valle Fértil are mostly ranchers.

### Surveys

Between 2010 and 2012 we interviewed 112 villagers older than 18 years of age, who are resident in six different locations within Valle Fértil. To cover a wide range of situations, we selected people from both sexes and with a different degree of involvement in ranching activities. Before being interviewed, local residents were briefed on the research project and its academic objectives, as suggested in the guidelines of the International Society of Ethnobiology Code of Ethics. After that, interviewees gave verbal informed consent in order to assure their anonymity. Semi-structured questionnaires were used, complemented by free interviews and informal conversations. Semi-structured forms were divided into two sections. First, we gathered overall socio-demographic information about surveyed residents. We recorded: gender, age, education level, occupation, place of residence, time of residence, and whether they currently have or ever had livestock (ranching). Then we focused on ranchers (residents who currently manage livestock or had in the past) and we collected information on their activity that included: ranching experience, type of livestock, location of livestock, herd size and management regime (see the explanatory variables in Table 1). We evaluated “perception” as the respondents’ opinion on whether they consider the condor a beneficial or an injurious species.

**Table 1:** Explanatory variables used to analyze people's perception on Andean condors.

Variable	Type of variable	Response	Details
**Gender**	Categorical	Man/Woman	
**Age**	Continuous (models)	18–77	
	Categorical (contingency tables)	• 18–39 • 40–59 • ≥60	
**Education level**	Categorical	• Elementary • High	Study level achieved by respondents at College
**Occupations**	Categorical	• Independent • Employee • Teacher Seller	Economic activity of respondents
**Place of residence**	Categorical	• Center • North • South • Hills • Another place	Place where respondents currently live
**Time of Residence**	Continuous (models)	1–75	Number of years living in the place
	Categorical (contingency tables)	• ≤10 • >10 • Always	
**Livestock Ranching**	Categorical	• Have or had livestock • Never had livestock	Relationship with livestock activity
**Ranching Experience**	Continuous (models)	1–60	Years of being a rancher
	Categorical (contingency tables)	• ≤10 • 11–20 • 21–30 • >30	
**Type of livestock**	Categorical	• Cows • Goat	
**Location of livestock**	Categorical	• Hills • Plains	Geographical location of livestock
**Head of Livestock**	Continuous (models)	1–150	Number of head of livestock
	Categorical (contingency tables)	• ≤49 • 50–100 • ≥101	
**Livestock management**	Categorical	• Low • Intermediate • Intense	Time and effort dedicated to livestock care

### Data analysis

We analyzed people's perception on the Andean condor using Binomial and Chi-Square tests. Fisher’s exact test was used when comparing groups which had less than five respondents. Generalized linear models (GLM) with binomial distribution of errors were used to identify the influence of social factors (gender, age, education level, occupation, place of residence, time of residence, livestock ranching) and ranchers’ characteristics (ranchers’ experiences, type of livestock, location of livestock, herd size and livestock care) on people's perception of condors. Separate models were fitted to the complete respondent dataset, and a sub-sample composed of ranchers solely, for these were the only ones who recognized having killed condors in response to livestock predation. Model selection was based on an Akaike information criterion (AIC) for small samples (AICc [57]). All analyses were carried out using GNU PSPP (version 0.8.2) and R software 3.0.1 [58]. We considered a statistical significance when *P*≤0.05.

## Results

### Socio-demography of surveyed people

The people surveyed mainly ranged between ages 40 and 59. Their education level was generally low and they had no formal job, with the exception of school teachers. Furthermore, most of respondents were engaged in livestock production (Table 2).

**Table 2:** Demographics of respondents.

Features	Category	N	Percentage %
**Gender**	Man	66	59,46
	Woman	45	40,54
**Age**	18–39	35	31,53
	40–59	54	48,65
	≥60	22	19,82
**Residence**	Center	62	55,86
	North	22	19,82
	South	12	10,81
	Hills	13	11,71
	Another place	2	1,80
**Education** **Level**	Elementary*	68	61,26
	High	21	18,92
	College	22	19,82
**Occupation**	Independent**	60	54,05
	Employed	38	34,23
	Teacher	8	7,21
	Student	3	2,70
	NA	2	1,80
**Livestock** **Relationship**	Have or had livestock	66	59,46
	Never had livestock	45	40,54

* Includes people without formal education

** Includes people like housewives, shopkeepers, retired, and tradesmen.

NA, unavailable data

### Social perception and attitude toward condors

Most people perceived condors to be detrimental (81.3%) rather than beneficial (18.7%, binomial test *P*<0.001). Most respondents (77.2%, *P*<0.001) claimed to have suffered livestock losses to condor attacks, but only 32.5% could assure witnessing those attacks, while the vast majority, 67.5%, had not actually seen the attacks but still attributed the losses to condors (binomial test *P* = 0.038).

A small number of people (14%) admitted to having hunted condors in response to the damage they cause. However, this number more than doubled (31%) when asked if they knew other people who currently hunt condors in the area, and reached 38.5% when asked if they knew people who had hunted condors at any stage in the past (X^2^ = 17.4, gl = 2; *P*<0.001). Finally, 64.1% of people suggested that hunting has led to the decline in the Andean condor population in the area, whereas 35.9% said that hunting had not any effect on this population. Nevertheless, most people (63%) did believe that condors deserve to be conserved (binomial test *P* = 0.013). The reasons behind this claim are mostly driven by condors being perceived as having an aesthetic and cultural value (84.8%) rather than by their ecological role (15.2%; X^2^ = 22.3, gl = 1; *P*<0.001). Most people agreed with the implementation of ecotourism projects related to condors, like birding, as a potential economic option for development of the area (84.62%).

### Effect of socio-demography on people perception of condors

**Fig 1:**
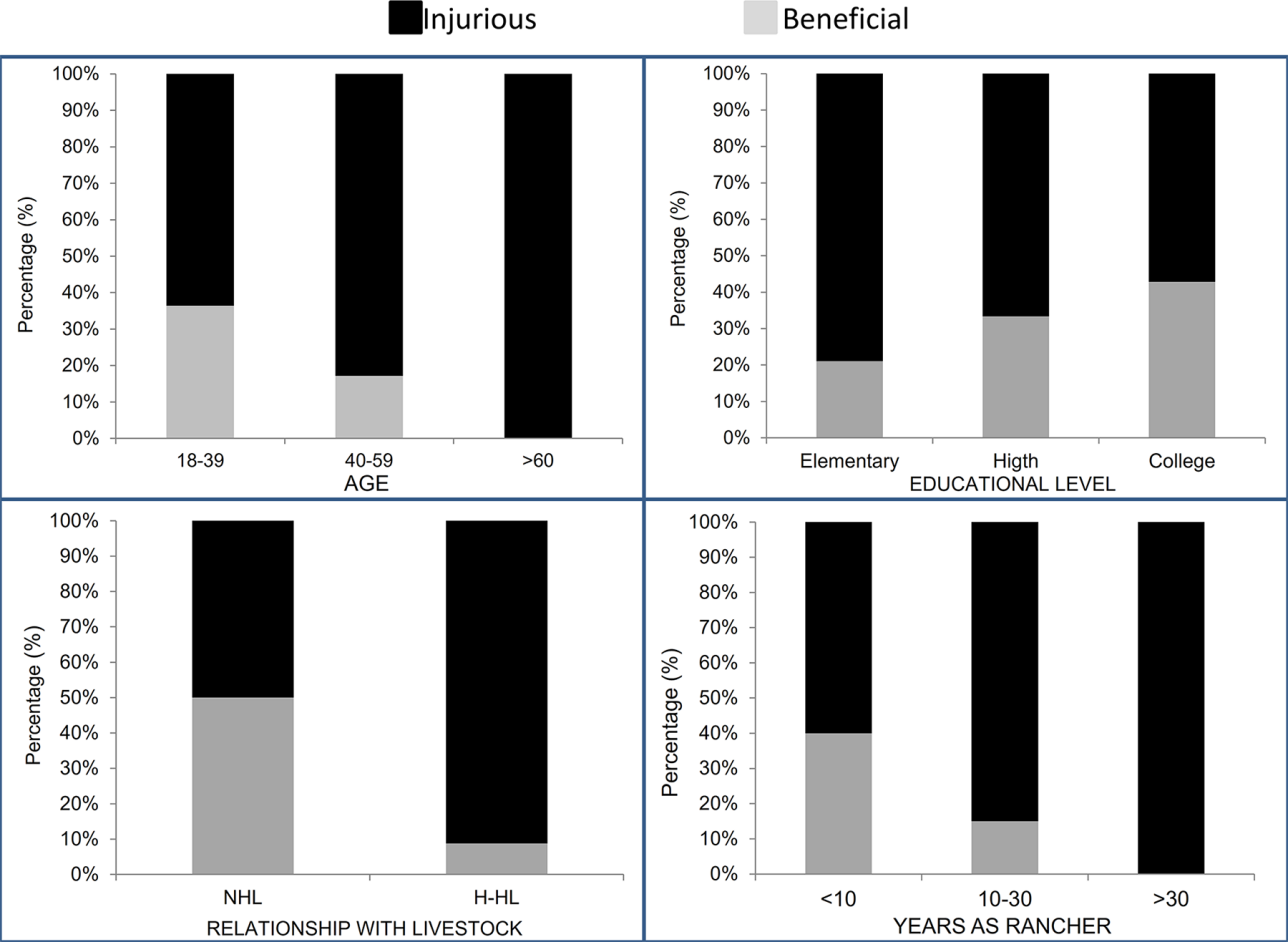
Perception of respondents (%) toward Andean condors split by social factors. A) Age, B) Education level, C) Livestock ranching (NHL, never had livestock; H-HL, have or had livestock) and D) Rancher’s experience.

The perception of condors differs significantly between genders. The proportion of men with negative perception (88.68%) is larger than that of women (63.64%) (X^2^ = 6.42, gl = 1; *P* = 0.011). Perception also varies in relation to age, reaching an overwhelming 100% of respondents with negative perception among people over 60 years of age (X^2^ = 8.40, gl = 2; *P* = 0.015, Fig 1A). Furthermore, even though most people perceive the condor as injurious, this species is being perceived as a beneficial species when education level increases (X^2^ = 8.40, gl = 2; *P* = 0.004, Fig 1B).

Place of birth was not clearly associated with perception of the condor (X^2^ = 9.486, gl = 5; *P* = 0.091), nevertheless, the negative perception reached 100% among people born at mountain sites. Moreover, the current place of residence was associated with a negative perception (X^2^ = 14.662, gl = 5; *P* = 0.012), with 90.9% of people who live in mountain areas having a negative perception. Negative perception was also associated with occupation (X^2^ = 12.796, gl = 3; *P* = 0.005). Most independent workers (96.3%) and employees (66.7%) had a negative perception of condors, whereas the percentage of teachers with negative perception was slightly lower (57.14%).

### Effect of livestock ranching on perception of condors

Ranching is a widespread activity in the area, with 69.3% of all respondents being somehow involved in ranching activities (N = 63). Within this group, 91.2% of people perceived the condor as a detrimental species, whereas that percentage decreased to 50% among people not engaged in ranching activities (X^2^ = 15.32, gl = 1; *P*<0.001, Fig 1C). Negative perception increased with ranching experience (X^2^ = 8.33, gl = 2; *P* = 0.016, Fig 1D). Surprisingly, type of livestock (X^2^ = 1.32, gl = 1; *P* = 0.250), location of livestock (X^2^ = 1.70, gl = 1; *P* = 0.193), herd size (X^2^ = 3.95, gl = 2; *P* = 0.139) and livestock care (X^2^ = 1.07, gl = 2; *P* = 0.586) were not associated with ranchers perception on condors.

### General models for social perception of condors

#### Model for all respondents

We built 128 GLM models to evaluate the effect of social factors (gender, age, education level, occupation, place of residence, time of residence, livestock ranching) on people's perception on condors. The best models for the complete dataset (all respondents) included the following explanatory variables: livestock ranching, education level, gender and occupation (Table 3). The variables with the highest relative importance (RI) were education level (0.98), occupation (0.89) and livestock ranching (0.72) for all people surveyed. The other variables had an RI value lower than 0.40 (Table 4).

**Table 3:** Generalized linear models with binomial distribution were used to test for factors that have a significant effect on people's perception of the Andean condor in Valle Fértil, San Juan, Argentina.

Model	Explanatory variable included	AICc	Δ Akaike	Weight	Percentage of explained deviance
**Models for Complete dataset (n = 112)**					
1	Livestock ranching + education level + occupation	59.06	0	0.23	50,78
2	Livestock ranching + education level + occupation + gender	60.74	1.68	0.1	51,74
**Models for Ranchers only (n = 90)**					
1	Ranching experience + education level	34.35	0	0.14	32,95
2	Education level	34.81	0.46	0.11	25,57
3	Education level + type of livestock	35.03	0.68	0.1	31,18
4	Ranching experience + education level + type of livestock	35.94	1.59	0.06	35,11
5	Ranching experience + education level + location of livestock	36.14	1.78	0.06	34,61

**Table 4:** Relative importance (RI) of explanatory variables used to explain social perception on the Andean condor for all people and for a subset of data composed only by ranchers.

Explanatory Variable	RI complete model	RI ranchers’ model
**Education level**	0.98	0.85
**Occupation**	0.89	
**Livestock Ranching**	0.72	
**Gender**	0.31	
**Time of residence**	0.27	
**Age**	0.25	
**Place of residence**	0.15	
**Ranching experience**		0.85
**Type of livestock**		0.4
**Location of livestock**		0.25
**Head of livestock**		0.25
**Livestock management**		0.09

A detail of the explanatory variables can be found in Table 1

### Model for ranchers

We analyzed only ranchers in order to assess the effect of ranchers’ characteristics (ranchers’ experiences, type of livestock, location of livestock, herd size and livestock care) on perception of condors, and to this end we built 64 GLM models. The explanatory variables included in the best models were: ranching experience, education level, type of livestock, and location of livestock (Table 3). The variables with the highest relative importance (RI) were education level (0.85) and ranching experience (0.85). The other variables had an RI value lower than 0.40 (Table 4).

## Discussion

In this work we study the nature of the conflict between people and condors in the central west of Argentina. In this area, condors are hunted or poisoned by ranchers and most people believe that these are the main causes of the condor population decline in the region in recent years. Worldwide, scavenging birds are in conflict with farmers due to livestock losses. For instance, Black Vultures (*Coragyps atratus*) are persecuted in some areas of the United States because of the damage they do to livestock [24,25,59]. For example, in South Africa, Cape Vultures (*Gyps coprotheres*) are threatened by traditional medicine use, but also by farmers [60], whereas European vultures are in conflict with humans mostly due to livestock predation [61,62]. After recovery of some vulture species, the conflict arose again and there possibly was a shift in the behavior of Griffon vultures (*Gyps fulvus*), whose attacks on livestock have been suggested to be a consequence of a lack of food [62]. Therefore, the human-scavenger conflict is a huge problem for vulture conservation.

To understand the complexities of the conflict we looked into people's perception on Andean condors. We identified a clear mismatch between the perception on condors by biologists and by local people who coexist with them, particularly those involved in ranching activities. From an ecological perspective, the Andean condor is at the top of the food chain and is considered an obligate scavenger who plays a fundamental role in cleaning the environment [47]. Yet, despite the relatively low percentage of people saying they had actually seen an attack (32.5%), most rural people believe that condors also actively prey on and kill livestock (81% of respondents). This is at odds with the scientific evidence on the species. Unlike for other scavenger species [24,25,59,62], there is still no systematic scientific evidence of condors attacking livestock, but there are some field observations on the possible predatory behavior of condor’s. Moreover, necropsies performed on lambs in Patagonia showed that predation by scavenging birds (including condors, vultures and caracaras) was never the primary cause of lamb death [63]. Instead, in the very few cases where predation did occur, the lambs showed signs of previous weakness (parasites, malnutrition, infections, others) that would have caused their death anyway [63]. Nevertheless, the conflict between humans and condors is a long-standing one and has been reported throughout the distribution range of the species [39,64]. Overall, ranchers attribute the death of newborn livestock to predation by condors and pumas, in similar proportions. The majority of people in Valle Fértil perceive the condor as an injurious species, and this negative perception has also been affected by different social factors, namely education level, occupation, livestock activities and ranching experience. However, education level, along with ranching activity, had the highest relative importance, there being a direct relationship between increased education level and a positive perception toward Andean condors. The relationship between perception and education is a common result in other studies that look into human-animal conflicts [12,65]. For instance, education influences knowledge of the ecological role of jaguars and pumas, and this, in turn, affects people's perception of these cats [66]. Furthermore, conservation of other natural resources such as soil and water also shows a strong relationship between perception and education [67–69].

Although gender was not included as an important variable in the models, it remain in one of the models selected. About gender and perception, an author [70] suggested that psychological structure is different in men and women and this affects their perception. He suggests that men view the world in more distinct, rational and logically differentiated ways, whereas women have a moral inclination to emphasize a highly articulated sense of responsibility, caring and compassion for others. In this context, some authors [71], in a job about attitudes, knowledge and behavior toward wildlife, concluded that gender is among the major demographic factors determining attitudes about animals. Females especially value wild animals as objects of affection but males are more inclined to value animals for practical and recreational reasons. These authors concluded that another factor affecting gender perception is education. For both, males and females, higher education is associated with more appreciation and a greater feeling of protection toward animals. This supports our results that indicate education as a most important factor affecting perception. In addition, a population’s cultural aspects can also account for the difference in perception between men and women. In rural societies, such as is our case, the roles for men and women are usually well differentiated. In this type of societies, the man is primarily in charge of providing for the family’s economic sustenance, and in many cases he is engaged in farming activities such as animal husbandry. The woman, instead, is devoted to home maintenance and childcare. These roles are maintained even for children, who learn the roles assigned to men and women from a very early age [44,72].

People's occupation was another factor we found to be associated with their perception of Andean condors. Not surprisingly, the vast majority of ranchers perceive them negatively. Nevertheless, we did not find a relationship between perception and some other factors like herd size or the place where they raise livestock, which had been suggested to play an important role in people's perception of jaguars in the South of Brazil [66].

It is important to highlight that, despite an overall negative perception, most interviewed people agreed that the condor must be conserved. This belief was even shared by half of the respondents who perceive condors as detrimental. The main reasons behind this statement are that condors are considered a local symbol, and that some people see an economic value in conserving this species, for instance by using condors as an attraction for ecotourism. Development of ecotourism projects related to condor sightings has already been suggested as a good alternative to change people's negative perception [73]. This would make it possible to balance the negative costs associated with condors conservation and the economic benefits that could be achieved from its use as an ecotourism resource [74]. However, for this strategy to be successful participation of local people in the revenue from ecotourism activities should be guaranteed [75]. Otherwise, the people’s present agreement on the notion that the condor must be conserved might disappear [74].

There is a need for general education programs for people, but particularly designed for ranchers, who are the ones in more conflict. Along these lines, some authors [76] suggest that the negative perception of condors may be reversed by highlighting their fundamental ecological role. Education programs target should be the recovering of the cultural value of the species [49]. Furthermore, it is important to determine, through scientific research, the existence of any damage from condors to livestock and, if damage does actually exist, then to quantify the demographic and economic impacts of such damage. This will help education programs to focus on working based on scientific data and will be useful in designing management strategies for ranchers.

Summarizing, we wish to highlight that Education level and Livestock ranching were the factors primarily affecting people’s perception of the condor. Nevertheless, Education is the only factor that could be modified through formal and informal programs. Therefore, it is important to take into account the human dimension of the issue, and work collaboratively with social researchers on developing integral and applicable management solutions to this human-wildlife conflict, in order to ensure Condor and other scavengers conservation.

## Supporting information

S1 Dataset All data underlying the findings reported. (XLSX)
